# A Novel Role for SIRT-1 in L-Arginine Protection against STZ Induced Myocardial Fibrosis in Rats

**DOI:** 10.1371/journal.pone.0114560

**Published:** 2014-12-12

**Authors:** Sherine M. Rizk, Shohda A. El-Maraghy, Noha N. Nassar

**Affiliations:** 1 Department of Biochemistry, Faculty of Pharmacy, Cairo University, Cairo, Egypt; 2 Department of Pharmacology and Toxicology, Faculty of Pharmacy, Cairo University, Cairo, Egypt; University of Central Florida, United States of America

## Abstract

**Background:**

L-arginine (L-ARG) effectively protects against diabetic impediments. In addition, silent information regulator (SIRT-1) activators are emerging as a new clinical concept in treating diabetic complications. Accordingly, this study aimed at delineating a role for SIRT-1 in mediating L-ARG protection against streptozotocin (STZ) induced myocardial fibrosis.

**Methods:**

Male Wistar rats were allocated into five groups; (i) normal control rats received 0.1 M sodium citrate buffer (pH 4.5); (ii) STZ at the dose of 60 mg/kg dissolved in 0.1 M sodium citrate buffer (pH 4.5); (iii) STZ + sirtinol (Stnl; specific inhibitor of SIRT-1; 2 mg/Kg, i.p.); (iv) STZ + L-ARG given in drinking water (2.25%) or (v) STZ + L-ARG + Stnl.

**Results:**

L-ARG increased myocardial SIRT-1 expression as well as its protein content. The former finding was paralleled by L-ARG induced reduction in myocardial fibrotic area compared to STZ animals evidenced histopathologically. The reduction in the fibrotic area was accompanied by a decline in fibrotic markers as evident by a decrease in expression of collagen-1 along with reductions in myocardial TGF-β, fibronectin, CTGF and BNP expression together with a decrease in TGF-β and hydroxyproline contents. Moreover, L-ARG increased MMP-2 expression in addition to its protein content while decreasing expression of PAI-1. Finally, L-ARG protected against myocardial cellular death by reduction in NFκ-B mRNA as well as TNF-α level in association with decline in Casp-3 and FAS expressions andCasp-3protein content in addition to reduction of FAS positive cells. However, co-administration of L-ARG and Stnl diminished the protective effect of L-ARG against STZ induced myocardial fibrosis.

**Conclusion:**

Collectively, these findings associate a role for SIRT-1 in L-ARG defense against diabetic cardiac fibrosis via equilibrating the balance between profibrotic and antifibrotic mediators.

## Introduction

Diabetes mellitus (DM) is projected to embrace 439 million by 2030 [1]. Noteworthy, cardiovascular diseases mount to three quarters of the deaths among population [2], [3]. Persistent hyperglycemia is pivotal in the incidence of diabetic cardiomyopathy (DCM), which is typically designated by increased cardiac cytokine [4], [5], inflammation, apoptosis as well as changes in the composition of the extracellular matrix (ECM) with enhanced cardiac fibrosis [2], [3], [5], [6].

Indeed, several studies have outlined the involvement of nuclear factor kappa (NF-κ)-B; a ubiquitous inducible transcription factor that activates a number of pro-inflammatory cytokines in DCM [7]–[10]. Furthermore, one of the profibrotic cytokines that stimulate ECM protein production is the transforming growth factor-β- (TGF-β); which in the heart, triggers cardiac fibroblasts to differentiate into the more active connective tissue cells known as myofibroblasts [11]. These myofibroblasts are capable of producing up to twice as much collagen as their fibroblast precursors [12]. In addition, TGF-β increases production of cellular adhesion molecules, which in turn increase myofibroblast survival and activity [13]. Noteworthy, in fibrosis, excessive ECM proteins alter myocardial structure, architecture and shape, which consequently affect cardiac function [14]. Nevertheless, the dynamic change in ECM is maintained via proteolytic enzymes, such as matrix metalloproteinases (MMPs), which are pivotal in promoting change and remodeling.

Silent information regulator (SIRT-1), a founding member of a large family of class III histone deacetylase, regulates a wide variety of cellular processes including cell cycle, and apoptosis [15], [16]. Noteworthy, SIRT-1 is highly expressed in murine embryonic heart where its knockout results in cardiac developmental defects. Furthermore, SIRT-1 is known to inhibit the transcriptional activity of NFκ-B [17], hence affecting many of its downstream mediators.

The immunomodulatory amino acid L-arginine (L-ARG) has been shown to possess an array of desirable biological properties [18]. Notably, elevated arginine levels in plasma correlate with changes in the secretion of various cytokines such as tumor necrosis factor-alpha (TNF-α) and hormones as insulin, which, in turn, may influence insulin sensitivity and glucose homeostasis [19]. Moreover, L-ARG has been shown to ameliorate complications of pulmonary hypertension [20], type-1 diabetes [21]as well as enhancing insulin sensitivity [22].

Although both SIRT-1 and L-ARG have been shown to separately modulate cytokine production and apoptosis, the implication of SIRT-1 in mediating the modulatory effect of arginine in ameliorating diabetic complications has not yet been investigated. To this end, the current study aimed at investigating the mechanism by which SIRT-1 mediates the protective effect of L-ARG in the absence or presence of the SIRT1 inhibitor, sirtinol (Stnl) against streptozotocin (STZ) induced myocardial fibrosis in rats.

## Methods

### Ethics statement

The current investigation conforms to the standard ethical procedures and policies approved by Ethical Committee for Animal Experimentation at Faculty of Pharmacy, Cairo University and were approved by the Guide for the Care and Use of Laboratory Animals published by the US National Institutes of Health (NIH Publication No. 85–23, revised 1996) [23].

### Animals

Adult male Wistar rats obtained from El Nile Pharmaceutical Company (Cairo, Egypt) weighing (200 ±20 g, 6–7 wks old) were used. Animals were allowed an acclimatization period for one week at the animal facility of the Faculty of Pharmacy, Cairo University (Cairo, Egypt). Rats were housed in groups at constant temperature (23±2°C), humidity (60±10%) and a light/dark (12/12 h) cycle. Access to food and water throughout the experimental period was allowed ad lib, unless otherwise specified.

### Experimental Design

A total of 120 animals were used in this study. Animals were allocated into 5 groups which were further divided into four subgroups (n = 6 animals per subgroup). The vehicle-treated group receiveda single intraperitoneal (i.p.) injection of 0.1 M sodium citrate buffer (pH 4.5) and served as controls (CONT). The other remaining groups were rendered diabetic by receiving a single (i.p.) injection of STZ (Sigma-Aldrich Chemical Co.; St. Louis, MO, USA) at the dose of 60 mg/kg dissolved in 0.1 M sodium citrate buffer (pH 4.5) [24]. Following the conformation of diabetes by Accucheck Active glucose strips (Roche Diagnostics Polska Ltd., Warszawa, Poland) using tail vein blood samples, only those rats with blood glucose levels>250 mg/dl 3 days after STZ injection were used. These animals either continued without treatment (STZ) and served as the diabetic rats, or receiving daily i.p. injection of sirtinol (Stnl; 2 mg/Kg) [25] dissolved in DMSO (Sigma-Aldrich Chemical Co.; St. Louis, MO, USA), started one week after induction of diabetes and continued for 7 weeks. L-ARG (Sigma-Aldrich Chemical Co.; St. Louis, MO, USA), given in drinking water containing 2.25% L-ARG and served as L-arginine treated group (STZ + L-ARG). L-ARG started one week after induction of diabetes and continued for 7 weeks. Group (STZ + Stnl + L-ARG) received daily 2 mg/Kg Stnl [25], in addition to the tap water containing 2.25% L-ARG. which started one week after induction of diabetes and continued for 7weeks. At the end of the experimental period, overnight fasted animals were sacrificed by decapitation and the blood was collected and divided on two aliquots; one was processed for serum preparation used for assaying the levels of glucose and insulin. The other was collected in heparinized tubes and used for the estimation of glycated hemoglobin (HbA1c%). The left ventricle of each animal was rapidly isolated, washed with ice-cold physiological saline and dried for the assessment of cardiomyopathy on biochemical as well as molecular levels. As mentioned previously, animals were divided into 4 subgroups for (i) histopathological staining (ii) hydroxyproline content, (iii) polymerase chain reaction (PCR) and flow cytometery and (iv) enzyme- linked immunosorbent assay (ELISA).

### Measurements of blood parameters

Fasting serum glucose (FSG) level was assayed enzymatically (Stanbio, San Antonio, TX, USA). Serum insulin levels were determined by ELISA (DRG International, New Jersy, USA), according to the manufacturers' instructions. HbA1c % was measured by HPLC using the kit supplied from Bio Rad D-10 (France) using the Bio Rad D-10 hemoglobin testing system [26].

### Assessment of total collagen contents using Masson'sTrichrome staining

Total collagen content was assessed by Masson'sTrichrome staining, as previously described [27]. Briefly, left ventricles were fixed in 4% formaldehyde in phosphate-buffered saline (PBS, pH = 7.35) for 24 h at 4°C and embedded in paraffin. Sections 4 µm were cut and deparaffinized then embedded in a Masson composition solution and light green silk fibroin solution to evaluate change in the interstitial fibrosis. Sections were assessed and quantified by digital image analysis using computer software scion image beta 4.03 (Scion corporation, USA).

### Hydroxyproline content

Total collagen content of the left ventricle was quantified biochemically by the hydroxyproline assay [28]. Fifty mg of left ventricle was mixed with 10 N HCl and was subject to tissue hydrolysis in oven at 120°C overnight. Hydrolysates were neutralized and mixed with chloramine T solution and oxidized for 20 minutes at room temperature. The oxidized product was reacted with p-dimethylaminobenzaldehyde in ethanol and H_2_SO_4_ solution at 60°C for 20 to 25 minutes. The resulting chromophore was quantified spectrophotometrically at 557 nm against a standard curve of known hydroxyproline concentration (0.6 to 15 g/ml).

### Measurement of TGF-β1, TNF-α and interleukin 1-β (IL-1β) and SIRT-1contents

Left-ventricular contents of TNF-α, TGF-β, IL-1 β and SIRT-1 were assayed using rat ELISA kits supplied by Labs Biotechnology Inc., Canada, WKEA MED supplies crop, New York, BioVendor Research and Diagnostic Products USA and Uscn Life Science, Inc, Wuhan, China, respectively according to the manufacturers' instructions.

### Assessment of Caspase (Casp-) 3 and metalloproteinase-2 (MMP-2) activities

The activity of left-ventricular Casp-3 was measured by using a colorimetric assay kit (Biosource International, California, USA). Briefly, the levels of the chromophore p-nitroanilide (pNA) released by Casp-3 activity in the tissue lysates were quantified spectrophotometrically at 405 nm following the manufacturer's instructions. The left-ventricular activity of MMP-2 was assayed using rat MMP-2 kits supplied by Boster Biological Technology Co., Inc., Femont, USA. All the procedures of the used kits were performed following the manufacturer's instruction manual. The protein content was measured according to the method of Lowry et al. (1951) [29].

### Flow cytometric analysis of Fas

Left ventricular tissue was minced and incubated in 0.25% collagenase H for 30 min at 37°C. Following the incubation, 0.25% trypsin-EDTA was added for 30 min at 37°C. Trypsin digestion was stopped by the addition of 1% fetal calf serum and washed with PBS. One million cells/100 µL were incubated with anti-Fas monoclonal antibody conjugated with phycoerythrine for 40 minutes in the dark. Then, cells were washed with PBS three times and the fluorescence intensity (an index for the percentage of Fas positive cells) was measured with the FACS Calibour (Coulter Epics XL, Beckman, USA).

### RNA extraction and real-time PCR analysis

Total RNA extraction from left ventricle was done using TRIzol (Invitrogen, Carlsbad, CA, USA) according to the manufacturer's instructions. The purity and concentration were determined spectrophotometrically at optical density of 260 and 280 nm before use. The optical density ratio at 260/280 nm ranged from 1.7 to 2.0. The isolated total RNA was reverse-transcribed into complementary DNA (cDNA) using the High Capacity cDNA Reverse Transcription Kit (Applied Biosystems, Foster City, CA) according to the manufacturer's instructions and all products were stored at −20°C. The expression of target genes were analyzed by qPCR using the SYBR Green PCR Master MIX (Applied Biosystems, California, USA) with the ABI PRISM 7000 sequence detection system (Applied Biosystems, Foster City, CA) and relative quantification software (Applied Biosystems, Foster City, CA). The sequences of the primers used are listed in Table 1. Glyceraldehyde-3-phosphate dehydrogenase (GAPDH) was used as the house-keeping gene. The thermal cycle protocol consisting of initial denaturation at 95°C for 10 min followed by 40 cycles with 30 s denaturation at 95°C and 30 s annealing/extension at 60°C. As a relative quantitation, fold changes were calculated following the 2^−ΔΔCt^ method. For each sample, the Ct value of target gene mRNA was normalized to the GAPDH endogenous control as ΔCT (ΔCT = Ct _target gene_ – Ct _GAPDH_). The fold change of the target gene mRNA in the experimental sample relative to control sample was determined by 2^−ΔΔCt^, where ΔΔCt = ΔCt _Experimental_ - ΔCt _Control_.

**Table 1:** The oligonucleotide primers sequence of studied genes and the housekeeping gene.

Accession number	Gene name	Forward primer	Reverse primer
M25297	BNP	5′-GTCAGTCGCTTGGGCTGT-3′	5′-AGAGCTGGGGAAAGAAGAGC-3′
NM_022266	CTGF	5′-GCTGACCTAGAGGAAAACATTAAGA-3′	5′-CCGGTAGGTCTTCACATGG-3′
NM_019143	Fibronectin	5′-CAGCCCCTGATTGGAGTC-3′	5′-TGGGTGACACCTGAGTGAAC-3′
M24067	PAI 1	5′-AGAGCCAATCACAAGGCACT-3′	5′-AGGCAAGTGAGGGCTGAAG-3′
NM_021578	TGF β 1	5′-CCTGGAAAGGGCTCAACAC-3′	5′-CAGTTCTTCTCTGTGGAGCTGA-3′
NM_007743	Collagen 1	5′-CCGTTGGCAAAGATGGTAGA-3′	5′-CTTGGTTAGGGTCAATCCAGTAG-3′
NM_ 008610	MMP 2	5′-GCTCTGTCCTCCTCTGTAGTTA-3′	5′-CCCTCCTAAGCCAGTCTCTATTA-3′
AK089660	NFκ-B	5′-GTCACCCATGGCACCATAAA-3′	5′-CAACCCTCAGCAAATCCTCTAC-3′
HW089298	SIRT-1	5′-CCCTCAAGTGCAGGGAGTAAAG-3′	5′-AGCCAAGGCTACACAAAGA-3′
NM_009810	Casp- 3	5′-AGCAGTGGTAGCGTACAAAGA-3′	5′-GATGGCTTGCCAGAAGATAC -3′
NM_007987	Fas	5′-AAGTCCCAGAAATCGCCTATG-3′	5′-TCTTGCCCTCCTTGATGTTATT-3′
NM_008084	GAPDH	5′-AACAGCAACTCCCACTCTTC-3′	5′-TGGGTGCAGCGAACTTTAT-3′

### Statistical analysis

The results were expressed as the mean ±SEM and statistical comparisons were carried out using one way analysis of variance (ANOVA), followed by Tukey's Multiple Comparisons test. The minimal level of significance was identified at P<0.05.

## Results

As DMSO served as a vehicle for Stnl, the effects of DMSO were investigated on the measured parameters in a pilot study. Since there was no difference between DMSO and control groups, only those for control are utilized in the current study.

### Effect of L-ARG on FSG, HbA1c% and insulin levels

STZ induced a robust increase in FSG and HbA1c% mounting to approximately 5 and 2folds, respectively compared to their control counterparts, with no significant difference between STZ and STZ + Stnl. Conversely, L-ARG significantly decreased FSG as well as HbA1c%valuesas compared to STZ and STZ + Stnl animals. Furthermore, animals receiving either STZ or STZ + Stnl showed marked decline in serum insulin, roughly by about 66% and 59% of control values, respectively, an effect that was reversed by L-ARG administration (Table 2). Notably, the effect induced by L-ARG on glucose homeostasis was surprisingly not reversed by Stnl co-administration.

**Table 2:** Effect of STZalone, or in combination with Stnl, L-ARG or L-ARG + Stnl on Fasting serum glucose (FSG), insulin and HbA1c%.

	FSG(mg/dl)	Insulin (ng/ml)	HbA1c%
**CONT**	89.2±5.8	0.95±0.1	4.1±0.5
**STZ**	480±38.3*	0.32±0.03*	9.5±1.3*
**STZ + Stnl**	523±42.7*	0.39±0.02*	9.8±0.08*
**STZ + L-ARG**	134.5±10.2^#ω^	0.74±0.09* ^# ω^	6.0±0.43* ^#ω^
**STZ + Stnl+ L-ARG**	145.5±11.8^#ω^	0.68±0.08* ^# ω^	5.73±0.7* ^#ω^

Data represent the means of sixexperiments ±SEM.

*^, #^ and ^ω^P<0.05 compared with CONT; STZ and STZ+ Stnl, respectively, using one-way ANOVA followed by Tukey's Multiple Comparison Test.

### Effect of L-ARG on STZ- induced myocardial histopathological changes, fibrotic area and hydroxyproline content

**Figure 1:**
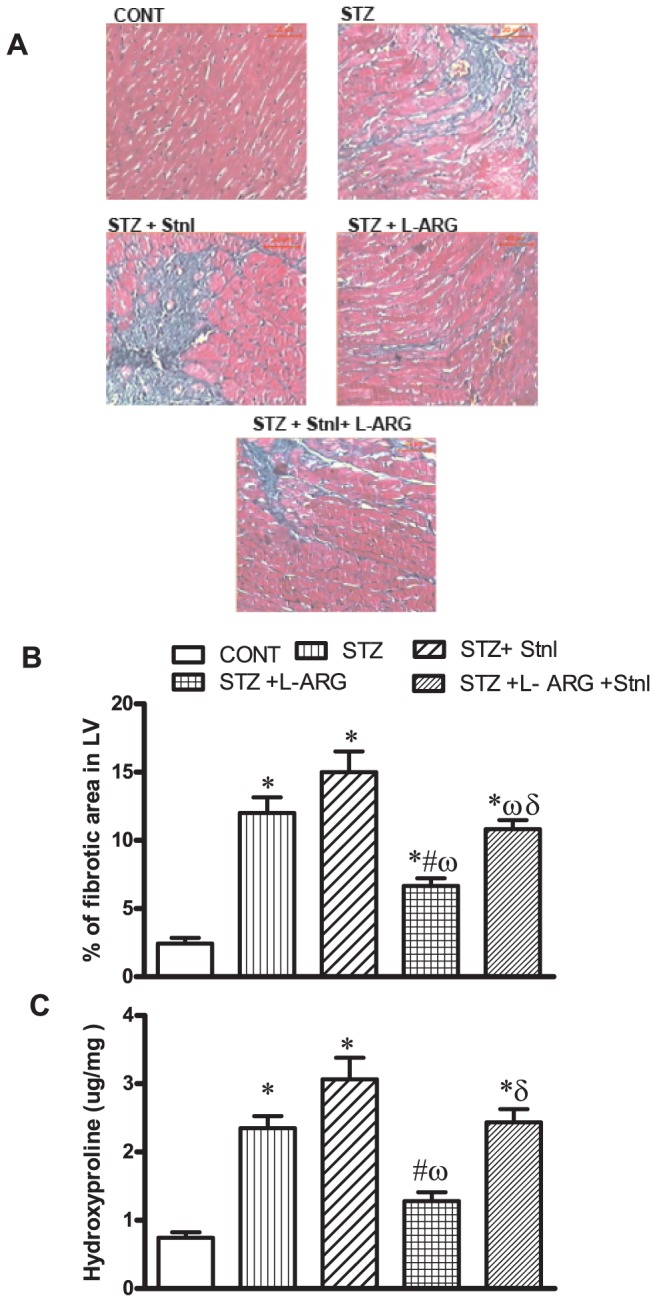
Effect of STZ alone or in combination with Stnl, L-ARG or Stnl+ L-ARG on incidence of fibrosis depicted histopathologically (A) and (B) % of fibrotic area in left ventricle (LV) as well as (C) LV hydroxylproline content. Data represent the means of six experiments ±SEM; *, ^#^, ^ω^ and ^δ^ P<0.05 compared with CONT, STZ, STZ + Stnl and STZ + Stnl + L-ARG, respectively, using one-way ANOVA followed by Tukey's Multiple Comparison Test.

Using Masson's Trichrome staining, severe fibrosis of the ventricular area in the STZ group (Fig. 1A). The ventricular area of fibrosis measured by Masson's Trichrome stain was significantly elevated in the STZ, STZ + Stnl and STZ + Stnl + L-ARG groups reaching 5.4, 6.2 and 4.2 folds of normal control values, respectively (Fig.1B). Such increase was significantly abrogated by L- ARG administration (Fig. 1B). By the same token, STZ diabetic animals as well as those receiving STZ + Stnl or STZ + Stnl + L-ARG showed3.15, 4.12 and 3.26 fold increase in hydroxyproline levels as compared to normal control values, respectively (Fig. 1C) which coincides with the marked increase in fibrotic area shown in these animals. On the other hand, animals receiving L-ARG displayed an obvious reduction in left ventricular hydroxyproline content (Fig. 1C) paralleling the improvement in ventricular fibrosis seen in that group.

### Effect of L-ARG on STZ -induced myocardial changes in fibrotic markers

**Figure 2:**
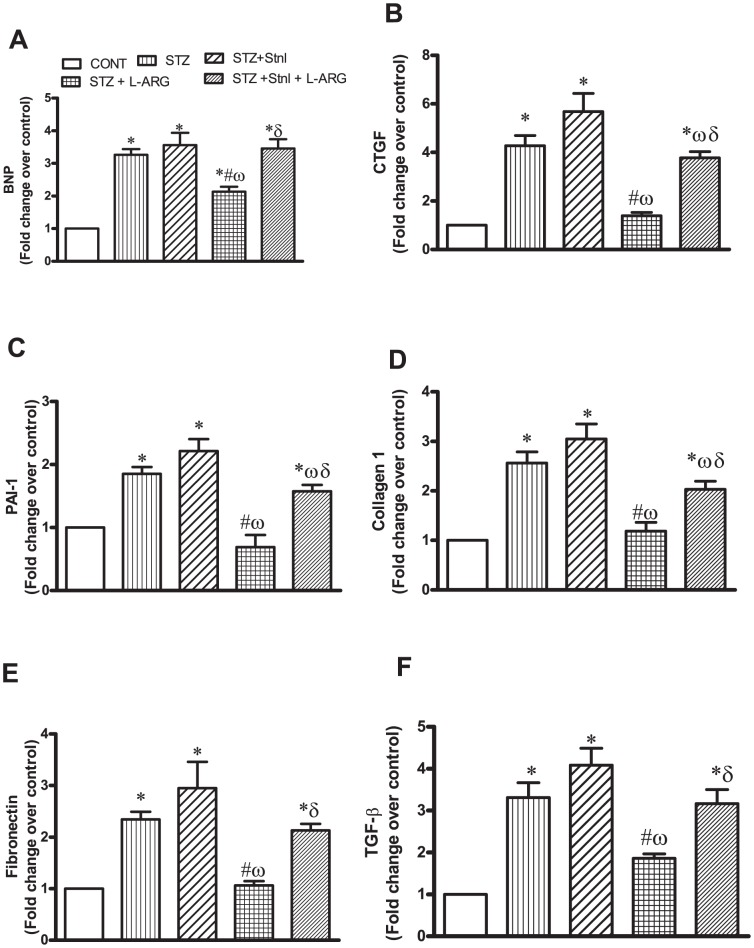
Effect of STZ alone or in combination with L-ARG on or L-ARG + Stnl: A-brain naturetic peptide (BNP), B- connective tissue growth factor (CTGF), C-Plasminogen activator inhibitor-1 (PAI-1), D-collagen-1, E-fibronectin and tansforming growth factor (TGF)-β. Data represent the means of six experiments ±SEM; *, ^#^, ^ω^ and ^δ^ P<0.05 compared with CONT, STZ, STZ + Stnl and STZ + Stnl + L-ARG, respectively, using one-way ANOVA followed by Tukey's Multiple Comparison Test.

Animals receiving STZ alone or along with either Stnl or Stnl + L-ARG showed a prominent increase in levels of mRNA for fibrotic markers manifested as brain naturetic peptide (BNP; 3.2, 3.6 and 3.5 folds, respectively), connective tissue growth factor (CGTF; 4.3,5.9 and 3.8folds, respectively), Plasminogen activator inhibitor (PAI-1; 1.8 and 2.2, 1.5folds, respectively), collagen-1 (2.6, 3 and 2 folds, respectively), fibronectin (2.4, 2.9 and, 2.1 folds, respectively) and TGF-β (3.3, 4.1 and 3.1folds respectively) compared to their respective normal controls (Figs. 2A–F). On the other hand animals given L-ARG showed a decline in all previous markers reaching 73%, 33%, 37%, 46%,45% and 53% for BNP, CGTF, PAI-1, collagen-1, fibronectin and TGF-β 1, respectively compared to those of STZ animals (Figs. 2A–F).

### Effect of L-ARG on STZ- induced myocardial changes in MMP-2 expression and protein content

Either STZ given alone or in combination with either Stnl or STZ+ Stnl + L-ARG induced reduction in both left ventricular MMP-2 mRNA and protein content compared to CONT animals. L-ARG given concomitantly with STZ increased MMP-2 at both expression and translational levels from STZ values (Fig- 3A, B).

**Figure 3:**
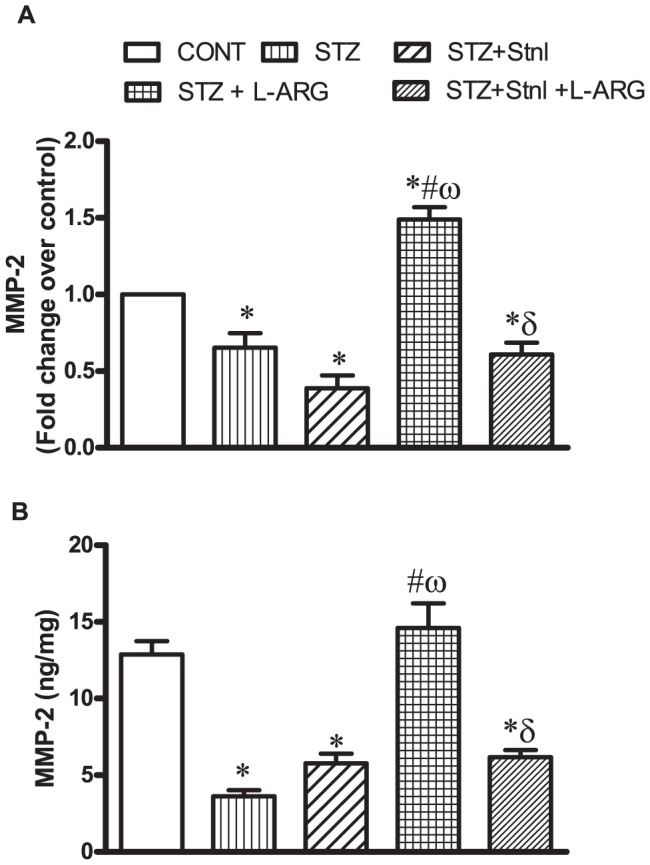
Effect of STZ alone or in combination with L-ARG or L-ARG + Stnl on metalloproteinase (MMP)-2 mRNA expression (A) and protein content (B). Data represent the means of six experiments ±SEM; *, ^#^, ^ω^ and ^δ^P<0.05 compared with CONT, STZ, STZ+ Stnl and STZ+ Stnl + L-ARG, respectively, using one-way ANOVA followed by Tukey's Multiple Comparison Test.

### Effect of L-ARG on STZ- induced myocardial changes in NFκ-B, TNF-α, IL-1β, TGF-β and SIRT-1

**Figure 4:**
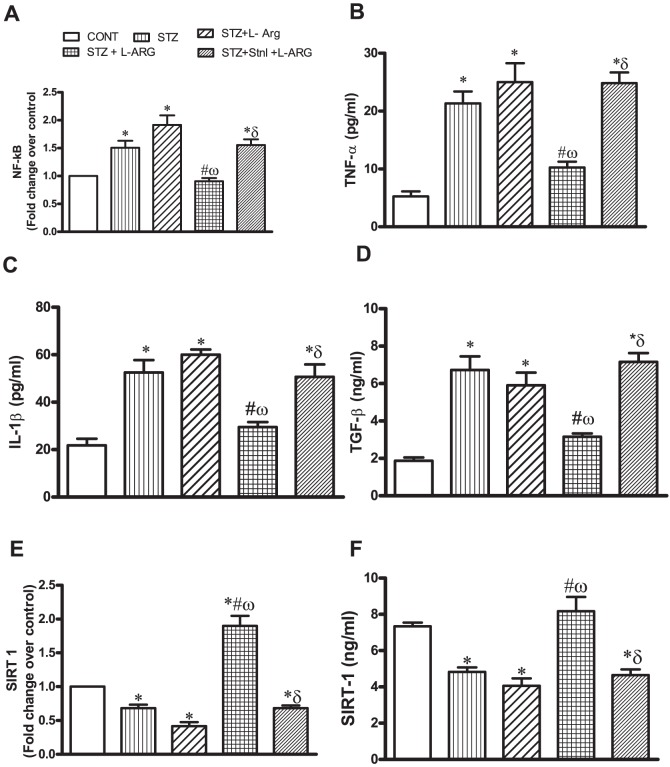
Effect of STZ alone or in combination with L-ARG or L-ARG + Stnl on (A) nuclear factor kappa (NFκ)-B mRNA expression, (B) tumor necrosis factor alpha (TNF-α) protein content, (C) interleukin 1 (IL-) β protein content, (D) silent information regulator (SIRT-1) mRNA expression and (E) its protein content. Data represent the means of six experiments ±SEM; *, ^#^, ^ω^ and ^δ^ P<0.05 compared with CONT, STZ, STZ + Stnl and STZ + Stnl + L-ARG, respectively, using one-way ANOVA followed by Tukey's Multiple Comparison Test.

Rats receiving STZ alone or in combination with either Stnl or STZ + Stnl + L-ARG showed marked increase in myocardial NFκ-B expression together with TNF- α protein, IL-1β and TGF-β contents. A marked decline in myocardial SIRT-1 mRNA was observed in the aforementioned groups by 32, 59 and 32%, respectively as well as its protein content compared to control values (Figs. 4A–F). Conversely, L-ARG given simultaneously with STZ decreased myocardial NFκ-B expression, TNF-α, IL-1β and TGF-β contents by 40, 52,49and 61%, respectively coinciding with observed increase in SIRT-1 expression by 2.6 folds as well as increased protein content by 1.7 folds compared to STZ animals (Figs. 4 A–F).

### Effect of L-ARG on STZ- induced myocardial changes in apoptotic markers

**Figure 5:**
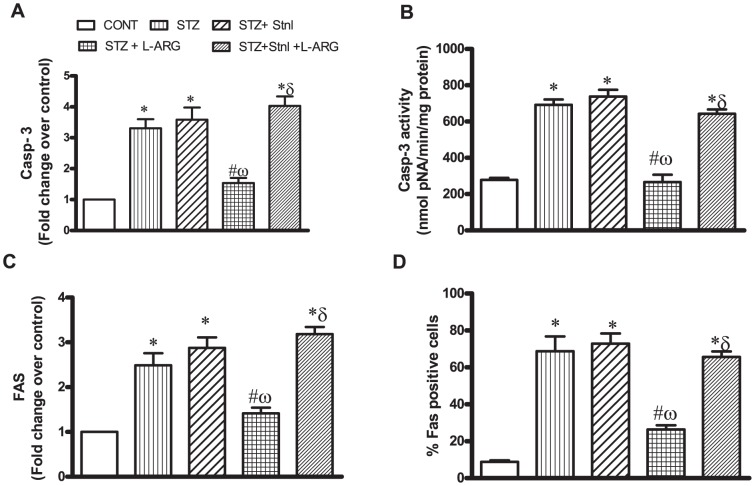
Effect of STZ alone or in combination with L-ARG or L-ARG + Stnl on caspase-3 (Casp-3) mRNA expression (A) and activity (B) as well as (C) FAS mRNA expression and (D) % FAS positive cells. Data represent the means of six experiments ±SEM; *, ^#^, ^ω^ and ^δ^ P<0.05 compared with CONT, STZ, STZ + Stnl and STZ + Stnl + L-ARG, respectively, using one-way ANOVA followed by Tukey's Multiple Comparison Test.

STZ diabetic rats produced significant apoptosis in left ventricular myocardial tissue as manifested by increased Casp- 3 and FAS mRNA by 3.3 and 2.5 folds, respectively compared to their respective controls, with no significant difference with STZ + Stnl or STZ + Stnl + L-ARG groups. By the same token, Casp-3 activity as well as the number of FAS positive cells were increased in STZ animals reaching to 249% 821% as compared to normal ones, respectively. On the other hand, L-ARG administered concomitantly with STZ normalized Casp-3 expression and activity as well as FAS expression. L-ARG significantly reduced the number of FAS positive cells as compared to STZ animals (Fig. 5).

## Discussion

Reported clinical and experimental data have provided compelling evidence indicating that either short- or long-term oral administration of L-ARG can improve insulin sensitivity [19]. Indeed, in the present investigation, L-ARG lowered FSG as well as HbA1c%, which is in line with previous findings [30], [31]. Intriguingly, animals given Stnl with L-ARG displayed no interference with the capacity of L-ARG to modulate FSG as well as HbA1c% (Table 2). However, the role for SIRT in mediating L-ARG regulation of diabetes sequela has not been fully elucidated. This assumption is further consolidated by the ability of Stnl to reverse the protective effects of L-ARG on fibrotic markers seen in the present investigation (Fig. 2). Noteworthy, SIRT- 1 intricately regulates many cellular processes including cell cycle, and apoptosis [15]. Indeed, in the present study, both SIRT-1 mRNA and protein content were decreased in STZ- rats and were restored following L-ARG (Fig. 4E-F). Conversely, Stnl, the SIRT-1 inhibitor, reduced L-ARG inducedSIRT-1 expression as well as its cellular content in diabetic rats (Fig. 4E-F). Furthermore, in the present study, L-ARG reduced NFκ-B expression compared to STZ insult (Fig. 4A). Notably, NFκ-B transcription can be induced by hyperglycemia [10]. This notion is further supported by current findings where animals receiving STZ as well as those give STZ + Stnl showed elevated FSG as well as HbA1c% (Table 2) thus positively associated with increased expression of NFκ-B. It is well known that SIRT-1deactylates NFκ-B hence tampering with its activity [17]. Notably, either SIRT1 inhibition or deletion concomitantly increased acetylation and NFκ-B [32]. Certainly, L-ARG by increasing SIRT-1 mRNA and protein content retarded NFκ-B expression. In further support for this notion, animals receiving Stnl in addition to L-ARG and STZ failed to show any decline in NFκ-B expression (Fig. 4A), a finding in line with a previous report [32]. Evidently, it has been reported that diabetes apart from presenting an altered metabolic condition, results from a perturbed immune regulation instigating inflammatory cytokines, e.g. TNF-α [33]. In the current study, animals receiving STZ either alone or with Stnl showed elevated left ventricular TNF-α as well as IL-1β levels in conjunction with elevated NFκ-B expression. L-ARG treatment reversed NFκ-B expression as well as TNF-αprotein content (Fig. 4A–C). Notably, activated NFκ-B translocates to the nucleus where it regulates the production of a plethora of proinflammatory cytokines, such as tumor necrosis factor [34], [35]. Blatantly, L-ARG enhances insulin sensitivity and ameliorates abnormalities of glucose metabolism in conjunction to attenuating production of inflammatory cytokines [19]. Furthermore, directly SIRT- 1 is known to modulate the functions of monocytes and macrophages [36], which are compromised in diabetes [37]. Such modulatory effects of SIRT-1 on proinflammatory cytokines have been shown to be inhibited by Stnl administration [38].

In addition, increased HbA1c % following DM generates free radicals and other advanced glycated end products (AGEs), consequently, evoking reactive oxygen species formation [39]. In DM, AGEs via sustained activation of NFκ-B [40] induces expression of inducible TNF-α [41]. Noteworthy, L-ARG has been shown to possess antioxidant properties [42] thus reducing AGEs [43] and hence NFκ-B as well as TNF-α[44]. This conception may provide a plausible explanation for the ability of Stnl to block L-ARG protective effects independent of modulating blood glucose and glycated Hb.

A multitude of clinical and experimental studies implicate elevated circulating glucose levels in cardiac inflammation and myocardial cellular dysfunction and death thus promoting fibrosis. Notably, Sklavounou et al. (2006) [45] reported that SIRT-1 is pivotal in preventing differentiation and apoptosis. Noteworthy, Stnl inhibition of SIRT1 enhances apoptosis [46]. In the current study, in addition to the elevated levels of TNF-α and IL-1β in conjunction with increased NFκ-B (Fig. 4A–C), Casp-3 as well as FAS mRNA and FAS positive cells were elevated (Fig. 5). The increased expression and production of Casp-3 indicates myocardial cellular death in association with STZ administration and STZ + Stnl. A plausible mechanism for the observed Casp-3 expression and hence myocardial cellular death may stem from the excessive TNF- α generation [47] as well as SIRT-1 inhibition. Accordingly L-ARG by down-regulating NFκ-B expression consequently halted TNF- α production and hence causing cessation of myocardial cellular death via reducing cardiac Casp-3 expression and protein content. Furthermore, Fas/FasL system constitutes a major contributor in the induction of apoptotic cell death [48]. In addition, the production of proinflammatory cytokines may be instigated by binding of FasL to Fas [49]. The action of Fas ligation may extend beyond its apoptotic activity, including NF-kB activation [50]. Such events lend further support to the observed increase in TNF-α and IL-1β in the current investigation in STZ animals, an event that was reversed by L-ARG administration and halted by Stnl implicating a role for SIRT-1 in mediating L-ARG effect against apoptotic injury.

Apoptosis has been reported to contribute to the loss of cardiomyocytes, an event that is followed by collagen deposition following activation of myofibroblasts thus replacing the space of damaged cardiomyocytes. Hence, fibrosis subsequent to apoptosis is acknowledged as a poor prognosis outcome in patients with diabetes. Moreover, increased NFκ-B expression is also known to activate TGF-β as well as fibronectin thus fostering increased ECM synthesis in diabetes [51]. Furthermore, one of the important steps in collagen formation is the hydroxylation of proline; hence, assessment of hydroxyproline level may give a clue about collagen accumulation of ECM. In the current study, animals receiving STZ and STZ + Stnl displayed increased myocardial hydroxyproline versus normalization in those treated with L-ARG (Fig. 1C). This notion might afford an explanation for the increased collagen deposition in the current investigation in animals receiving STZ. However, those given L-ARG showed increased SIRT-1 expression parallel to decreased NF-κB, which reflected as decreased production of profirotic markers manifest as TGF-β as well as fibronectin (Fig. 2). These events were held in check by Stnl concomitant administration with L-ARG to STZ animals thus interrupting SIRT-1 induced L-ARG protection against fibrosis. The current investigation outlines an increase in BNP expression in STZ animals signifying an important marker of fibrosis [52]. This increment was reduced by L-ARG administration (Fig 2A).

CTGF, another potent pro-fibrotic protein is reported to mediated tissue and organ fibrosis thus contributing to structural and functional abnormalities in the diabetic heart [53], [54]. Notably, CTGF expression is increased in type I DM and is accompanied by increased expression of fibronectin and collagen type 1 [55]. Indeed, in the current investigation, STZ, STZ + Stnl or STZ + Stnl+ L-ARG animals displayed increased CGTF expression, which was reversed by L-ARG treatment (Fig. 2B). Noteworthy TGF-β induced activation of fibroblasts, thus increasing collagen expression in the heart [56]. However, CTGF independently may activate fibroblasts and increase collagen-1 expression in the heart [57]. TGF- β and CTGF have been found in in cultures of rat cardiac fibroblasts, in the myocardium of spontaneously hypertensive rats and in the kidney of L-nitroarginine methyl ester hypertensive rats [58]–[62]. Another important contributor to fibrosis is the PAI-1, a protease inhibitor crucial for down-regulation of plasmin and fibrin proteolysis [63]. PAI-1 expression is induced in response to many factors including proinflammatory cytokines such as TNF-α [64]. Furthermore, the transcription factor NF-κB is central in the induction of a chronic inflammatory state associated with diabetes, [64]. Accordingly, in the current investigation, the induction of TNF-α via activation of NFκB, offers a mechanistic explanation for the observed increase in PAI-1 in STZ animals. On the other hand, L-ARG administration to STZ animals reduced PAI-1 expression (Fig 4C). A plausible account for this reduction stems from a decline in TNF-αconsequent to moderation of NF-κB expression. Furthermore, it is empirical to implicate a role for SIRT-1 in mediating L-ARG action via its inhibitory action on NF-κB expression. Such notion is further consolidated by the lack of protective effect of L-ARG on fibrotic markers when co-administered with Stnl. The observed changes in profibrotic mediators seen with STZ insult and ameliorated by L-ARG treatment were reflected as increased and decreased fibrotic positive areas, respectively upon histological examination (Fig. 1A-B).

Interestingly, the enhanced production of collagen is naturally accompanied by inherent defense mechanisms where the body breaks down the formed collagen to circumvent generation of fibrotic nonfunctional regions. Among these mechanisms MMPs expression is central in degradation of collagen [65]. Indeed cardiovascular disorders are associated with decreased expression of MMP-2 [66], which has been shown to be a direct mediator of cardiac fibrosis in diabetic cardiomyopathy [67]. Our results, demonstrated that animals given STZ as well as those receiving STZ + Stnl, MMP-2 expression as well as its cellular content were decreased, while L-ARG treatment modulated both its the expression and content (Fig. 3). A plausible mechanism for L-ARG protection may stem from its ability to induce SIRT-1 expression and content, as confirmed by lack of protection with L-ARG when given along with Stnl to STZ animals. Notably, SIRT-1-mRNA induction and the increment in its content inhibited NFκB expression as well as TNF-α, events that are observed in the current study. Noteworthy, NFκB has been shown to directly inhibit MMP-2 expression [68].

## Conclusions

This is the first study implicating the role for SIRT-1 in L- ARG defense against diabetic cardiac fibrosis via equilibrating the balance between profibrotic and antifibrotic mediators. It is imperative to note although L-ARG induced modulation against increased FSG and glycated Hb, its profibrotic and antifibrotic effects are not just dependent on glucose homeostasis. This finding is consolidated by the ability of Stnl to reverse L-ARG protection against fibrotic injury albeit modulating glucose homeostasis.
